# Quantity and Size of Titanium Particles Released from Different Mechanical Decontamination Procedures on Titanium Discs: An In Vitro Study

**DOI:** 10.3390/dj12050123

**Published:** 2024-04-24

**Authors:** Anthony Kao, Andrew Tawse-Smith, Sunyoung Ma, Warwick J. Duncan, Malcolm Reid, Momen A. Atieh

**Affiliations:** 1 Sir John Walsh Research Institute, Faculty of Dentistry, 310 Great King Street, Dunedin 9016, New Zealand; anthonyks@hotmail.com (A.K.); sunyoung.ma@otago.ac.nz (S.M.); warwick.duncan@otago.ac.nz (W.J.D.); momen.atieh@mbru.ac.ae (M.A.A.); 2 Department of Chemistry and Centre for Trace Element Analysis, Department of Geology, Dunedin 9054, New Zealand; malcolm.reid@otago.ac.nz; 3 Department of Oral Diagnostics and Surgical Sciences, Hamdan Bin Mohammed College of Dental Medicine, Mohammed Bin Rashid University of Medicine and Health Sciences, Dubai P.O. Box 505055, United Arab Emirates; 4 School of Dentistry, University of Jordan, Amman 11942, Jordan

**Keywords:** dental implants, peri-implantitis, instrumentation, titanium, scanning electron microscopy, inductively coupled plasma mass spectrometry

## Abstract

Complications such as peri-implantitis could ultimately affect the survival of a dental implant. The prevention and treatment of peri-implant diseases require managing bacterial biofilm and controlling environmental risks, including the presence of pro-inflammatory titanium (Ti) particles in the peri-implant niche. Objectives included the evaluation of the size and quantity of Ti particles released from moderately roughened Ti surfaces during common mechanical surface decontamination methods. One hundred and forty moderately roughened Ti discs were divided into seven groups (n = 20 per group); six groups received mechanical decontamination procedures (ultrasonic scaling (US) with a metal tip and poly-ether-ketone (PEEK) under low and medium power settings, air-polishing with erythritol powder, and Ti brush), and the control group underwent air–water spray using a dental triplex. The rinsing solution was collected for Ti mass analysis using inductively coupled plasma mass spectrometry (ICPMS), as well as for Ti particle size and count analysis under scanning electron microscopy (SEM) with energy-dispersive spectroscopy (EDS). US metal tip instrumentation generated 34.00 ± 12.54 μg and 34.44 ± 6.08 μg of Ti under low and medium power settings, respectively. This amount of Ti generation was significantly higher than other instrumentation methods. The mean Ti particle size of the US groups ranged from 0.89 ± 0.27 μm to 1.25 ± 0.24 μm. No statistically significant difference was found in the particle size among US groups and Ti brush group (1.05 ± 0.11 μm), except for US with the PEEK tip, where a significantly smaller mean particle diameter was found at the low power setting (0.89 ± 0.27 μm). Mechanical instrumentation can produce Ti particulates and modify the implant surfaces. US using a metal tip generated the highest amount of Ti with smaller Ti size particles compared to all other commonly used mechanical surface instrumentations. The EDS analysis confirmed Ti in PEEK US tips. It can be suggested that deterioration from the PEEK US tip and Ti brush, as observed under SEM, is an additional source of Ti release during Ti surface decontamination.

## 1. Introduction

Dental implants are widely accepted as the treatment of choice in rehabilitating the masticatory function and aesthetic concerns for patients with missing natural teeth. To achieve a successful long-term clinical outcome, it is important that the osseointegration is maintained and the changes in the marginal bone levels are minimal. In complications such as peri-implantitis, tissue inflammation causing loss of integrated bone surrounding the implant [1,2,3], could ultimately affect the survival of the implant. Recent systematic reviews of the epidemiology of peri-implant disease have shown a wide range of prevalence of peri-implantitis (1% to 47%), with a mean prevalence of 22% [4,5,6,7]. Peri-implant bone loss shares a similar immunopathogenesis with periodontitis yet presents some heterogeneities in the presentation. Biopsy of soft tissue with peri-implantitis and the analysis of soft tissues from retrieved implants have shown a large number of inflammatory cell infiltrates with a higher portion of neutrophil granulocytes and macrophages [8,9].

The prevention and treatment of peri-implant diseases requires management of bacterial biofilms and controlling environmental risks such as smoking and uncontrolled diabetes [10,11]. Meticulous personal oral hygiene and regular plaque control performed by a dental professional are fundamental in disease prevention [12,13]. Although there is no gold-standard protocol in managing bacterial biofilm on dental implants, no other methods are more effective than mechanical decontamination [14,15,16]. There has been much attention on assessing the efficacy of biofilm removal and the adverse effects of commonly applied mechanical instrumentation such as ultrasonic scaling, air-polishing, and rotatory brushes on titanium Ti surfaces. Ti surface alteration, in conjunction with Ti particulate generation, has been reported in the literature [17,18,19].

Studies on aseptic bone resorption in orthopaedic joint replacement have shown an upregulation of pro-inflammatory cytokine production as well as hard and soft tissue destruction in the presence of Ti particulates with a diameter below 10 μm [20,21,22,23,24,25]. A similar finding was observed for dental implants [19]. The expression of receptor activator of nuclear factor kappa-B ligand (RANKL) and inflammatory cytokines was significantly higher in areas containing Ti particles within peri-implantitis soft tissue biopsy [26]. Furthermore, the presence of Ti particles, together with lipopolysaccharides (LPSs) from gram-negative bacteria, demonstrated a synergistic activation of pro-inflammatory cytokine production by stimulated macrophages [27,28,29].

It is currently accepted that the pro-inflammatory response and cytotoxic potential of Ti particles in the peri-implant tissue at the cellular level is related to its quantity and physicochemical properties [30,31,32]. It is therefore important to understand the characteristics of the Ti particles that are generated as a result of ongoing plaque control procedures, as well as the treatment of peri-implant diseases. The aim of the in vitro study that is the subject of this research project was to analyse the quantity and size of Ti particulates that were generated from a moderately roughened Ti surface by commonly applied mechanical implant surface decontamination procedures.

## 2. Materials and Methods

### 2.1. Ti Discs and Group Allocation

**Figure 1:**
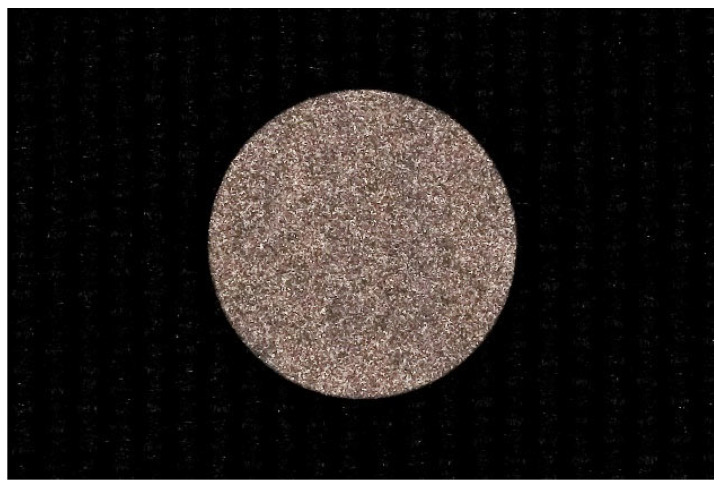
Commercially pure Grade 4 moderately roughened titanium disc.

Commercially pure Grade 4 moderately roughened (Sa = 1.34 and Ra = 2.14 μm) Ti discs (ø 10.0 mm × 3 mm) (Southern Implants, Irene, South Africa) (Figure 1) were used to simulate the Ti implant surface in this study. A total of 140 Ti discs were randomly assigned into seven groups (n = 20) using an online random number generator (www.random.org, accessed on 1 April 2024). The determination of the sample size needed was based on the amount of titanium particles generated by ultrasonic scaling, as data on the effects of other methods of decontamination are scarce. To detect a mean difference of 0.5 μg (standard deviation: 0.8 μg) using a power of 95%, and a two-sided significance level of 5%, a sample size of 140 titanium discs was required. The difference was based on a study conducted by Tawse-Smith and colleagues in 2016 [18]. The sample size calculation was performed using statistical software (Gpower software, Version 3.1.9.4). Six groups received six different mechanical instrumentation techniques with one control group:

(1) Ultrasonic scaling with a metal tip at a low power setting;
(2) Ultrasonic scaling with a metal tip at a medium power setting;
(3) Ultrasonic scaling with a PEEK tip at a low power setting;
(4) Ultrasonic scaling with a PEEK tip at a medium power setting;
(5) Air-polishing with erythritol powder;
(6) Ti brush only;
(7) Active control group: Rinsing with air–water spray using a dental triplex only.

Each group was then divided into two subgroups of 10; the rinsing solution of each subgroup was collected for the following:

Subgroup (a) Trace elements were analysed by inductively coupled plasma mass spectrometry (ICP-MS).
Subgroup (b) Ti particle size was measured with a scanning electron microscope (SEM) with the aid of energy-dispersive X-ray spectrometry (EDS) for particle differentiation.

**Figure 2:**
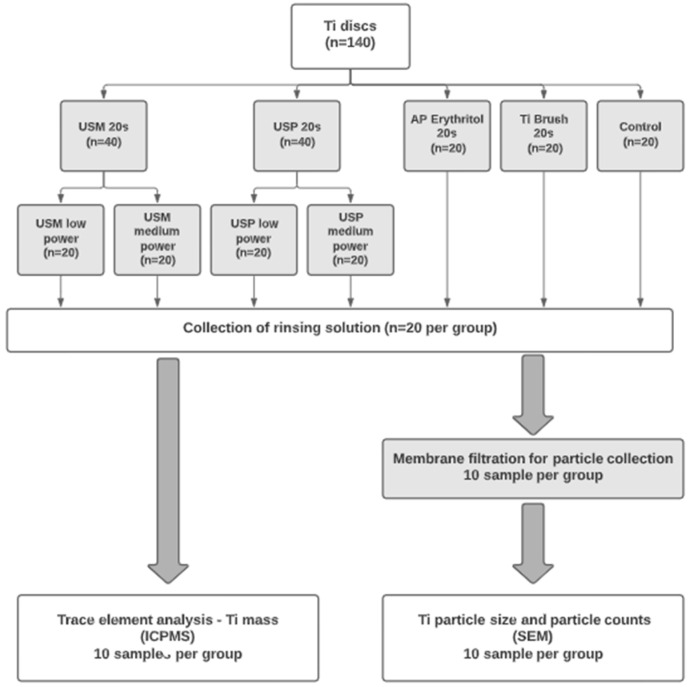
Study outline and group allocation. Note: Ti: titanium; USM: ultrasonic scaling with metal tip; USP: ultrasonic scaling with PEEK tip; AP: air-polishing with erythritol powder; ICP-MS: inductively coupled plasma mass spectroscopy; SEM: scanning electron microscope.

Figure 2 displays this diagrammatically.

### 2.2. Mechanical Instrumentation on Ti Surface

#### 2.2.1. Ultrasonic Scaling–Metal and PEEK Tip

**Figure 3:**
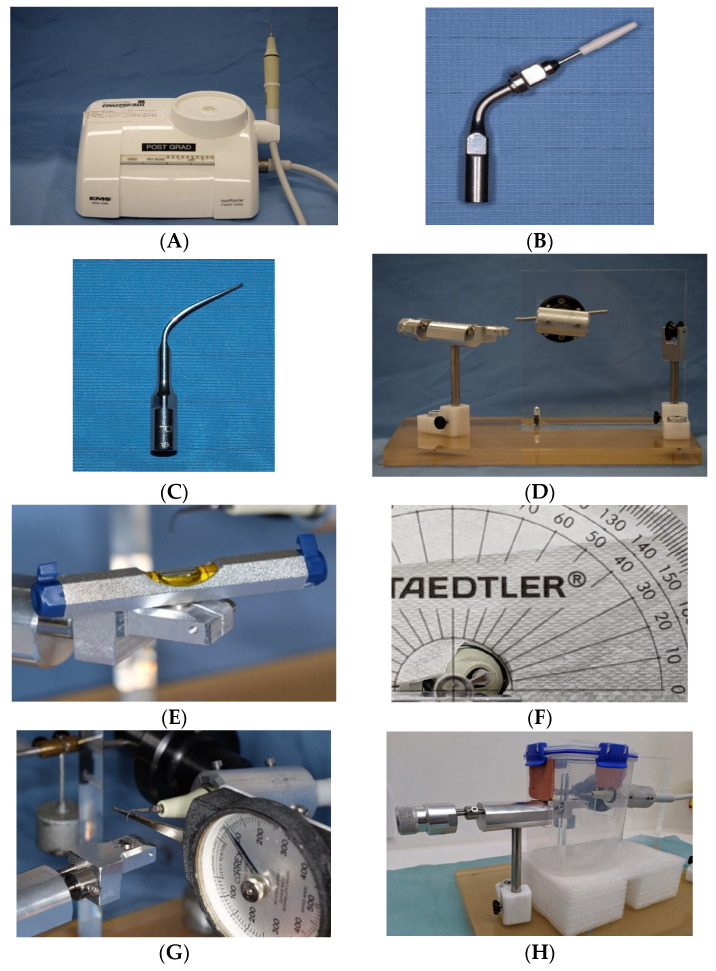
Setup of ultrasonic scaling with metal and PEEK tips: miniMaster piezon scaler E.M.S. (**A**), steel tip P, E.M.S. (**B**), PEEK Instrument PI, E.M.S. (**C**), custom instrumentation device (**D**), horizontal disc platform (**E**), scaling tip fixed at 15° (**F**), force controlled at 100 g (**G**), and enclosure for rinsing solution collection (**H**).

The ultrasonic scaling unit (miniMaster Piezon scaler^®^, E.M.S. Electro Medical System S.A., Nyon, Switzerland) with steel tip P (DS-011A, E.M.S.) and a PEEK instrument PI tip (DT-O65A, E.M.S) (Figure 3A–C) were used to instrument the Ti discs. Two groups were assigned to one of these two ultrasonic scaling instruments and then received ultrasonic scaling at either a low power setting (3 lights, 2.4 watts) or a medium power setting (5 lights, 3.8 watts), as recommended by the manufacturer for both scaling tips. The reservoir of the device was filled with standardised MilliQ solution used for irrigation during ultrasonic scaling. The dispensing volume of the MilliQ solution was calibrated and controlled at 3.0 to 4.0 mL for 20 s. To further standardise the instrumentation procedure, the Ti disc was placed on a horizontal sliding platform of a custom-made instrumentation device (Figure 3D,E). A metal jig on the instrumentation device held the scaler handpieces in place, the working end of the scaler was angled, and a protractor measurement fixed it at 15° to the disc surface (Figure 3F). Free weights (100 g) calibrated by an orthodontics dial tension meter provided the force exerted from the scaling tip onto the Ti disc (Correx Haag-Streit Tension meter, Haag-Streit AG, Korniz, Switzerland) (Figure 3G). A transparent sealed container was modified to enclose the instrumentation field and collect the rinsing solution (Figure 3H). The ultrasonic scaler was activated for 20 s continuously, while Ti disc slid back and forth across its diameter against the scaling tip at one stroke per second. The rinsing solution was collected in the container and then transferred to a 50 mL Digitube (AnalytiChem Inc, Baie D’Urfé, QC, Canada) using a 6 mL luer-lock syringe. The Ti disc, disc platform, the tip of the metal scaler, and the inner surface of the container were then rinsed with MilliQ solution for 10 s (approximately 15 mL) to retrieve the remaining free particles for sample collection. One tip was used for ten discs for both the metal and PEEK groups.

#### 2.2.2. Air-Polishing

**Figure 4:**
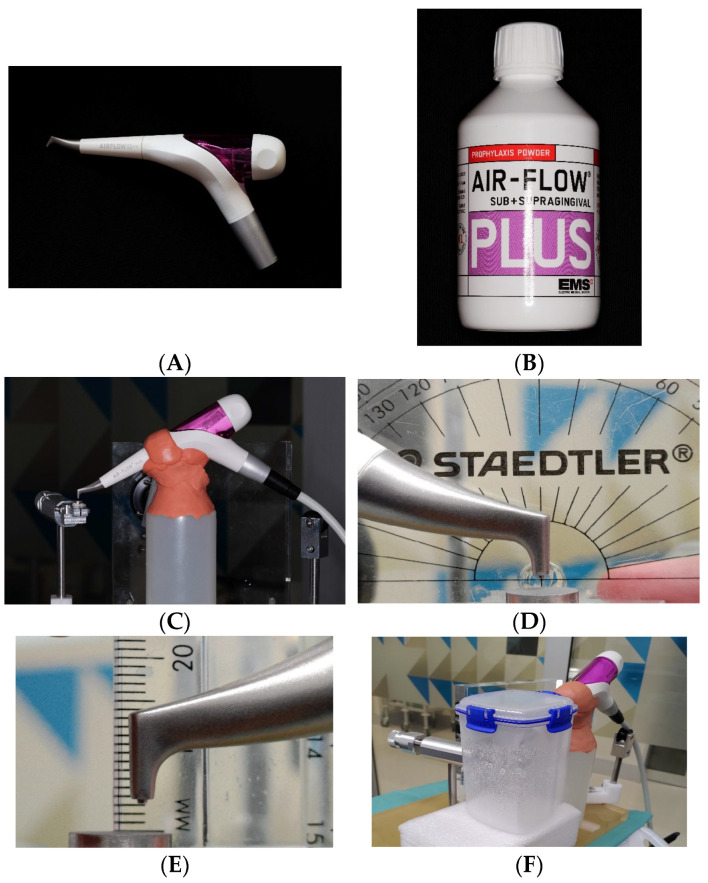
Setup of air-polishing: Air-Flow handy 3.0^®^, E.M.S. (**A**), Air-Flow Plus erythritol powder, E.M.S. (**B**), air-polish setup on instrumentation device. (**C**), nozzle fixed at 90° (**D**) and 3 mm away from the disc (**E**), and solution collected within a transparent enclosure (**F**).

Two groups of 10 discs received air-polishing treatment using an Air-Flow handpiece with a supragingival nozzle (Air-Flow handy 3.0^®^, E.M.S. Electro Medical System S.A., Nyon, Switzerland) (Figure 4A). The portable air-polishing device was connected to the turbine connection (Dentsply Sirona Sinius, Sirona Dental System GmbH, Bensheim, Germany). The water and air pressure delivered by the turbine connection were recorded at 2.2 and 4.7 bar, respectively. The water volume was adjusted and calibrated at an average of 24.4 mL per 20 s. Erythritol-based powder formula containing 0.3% chlorhexidine (Air-Flow Plus^®^, E.M.S. Electro Medical System S.A., Nyon, Switzerland) (Figure 4B) with an average particle size of 14 μm was used in conjunction with this setup. Twenty seconds of air-polishing was conducted with the nozzle fixed 3 mm away from the Disc at a 90° angle (Figure 4D,E) to the horizontally positioned Ti disc held in place on the previously described platform (Figure 4C). Ten seconds of post-instrumentation rinse (15 mL) was carried out as previously described for ultrasonic groups to complete the sample for collection.

#### 2.2.3. Ti Brush

**Figure 5:**
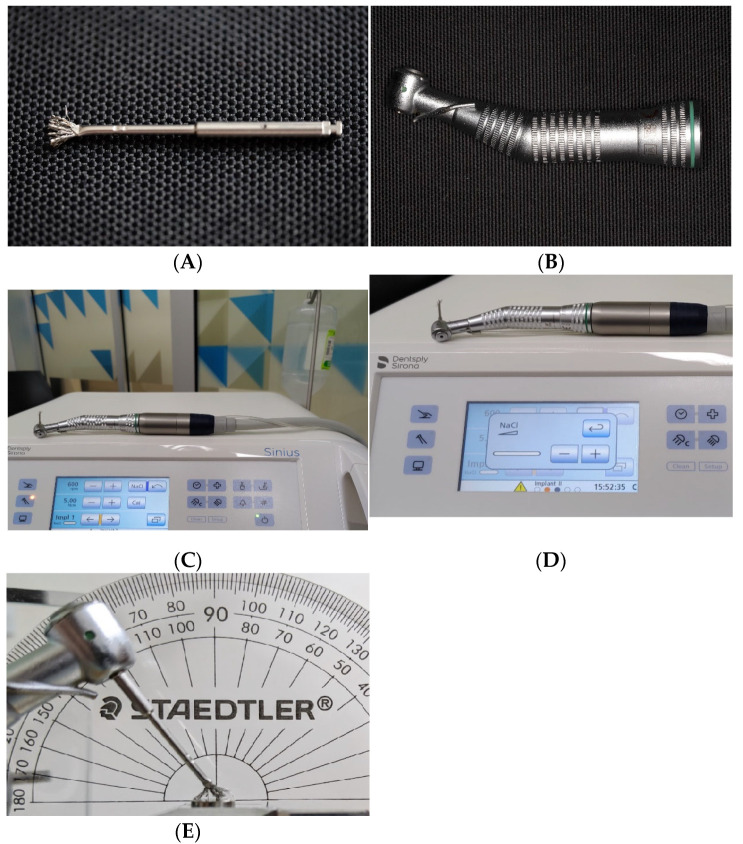
Setup of Ti brush: NiTi Brush Omega, Hans Korea (**A**), KaVo 16:1 Intra 3624N contra-angle surgical handpiece (**B**), setting of implant electric motor (600 RPM and 5Ncm torque (**C**), irrigation volume control (**D**), and Ti brush fixed at 55° the disc (**E**).

A nickel–titanium (NiTi) brush (NiTi Brush Omega, Hans Korea, Gyeonggi-do, Republic of Korea) was used in conjunction with a 16:1 contra-angle surgical handpiece (KaVo Intra 3624N, KaVo Dental GmbH, Biberach, Germany) (Figure 5A,B). The revolutions per minute (RPM) and torque setting on the integrated implant electric motor (BL implant electric motor, Sirona Dental System GmbH, Bensheim, Germany) were set at 600 RPM and 5 Ncm, respectively (Figure 5C). The 16:1 surgical handpiece therefore delivered a resultant 750 RPM that fell within the manufacturer’s recommendation of 600–1200 RPM. The irrigation volume was set to the minimum that provided around 10.6 mL of the solution in 20 s (Figure 5D). The MilliQ solution was delivered to the handpiece via an external bottle with the quantity controlled by the integrated pump of the implant electric motor. The instrumentation was performed on the custom device, and the rinsing solution was collected as previously described for the ultrasonic scaling groups (i.e., 10 s 15 mL). The shaft of the NiTi brush was fixed at 55° in relation to the horizontally placed Ti disc and delivered 50 g of force against the instrumented surface (Figure 5E). A new single-use NiTi brush was used for each disc instrumentation.

#### 2.2.4. Control

Two groups of 10 discs that served as controls were rinsed for 20 s using an air–water spray (triplex) that delivers MilliQ solution from the reservoir at 350 kPa. The tip of the triplex was held 2 cm away from the Ti surface in a perpendicular direction.

### 2.3. Particle Analysis—Scanning Electron Microscopy

#### 2.3.1. Sample Preparation for SEM—Particle Filtration and Collection 

**Figure 6:**
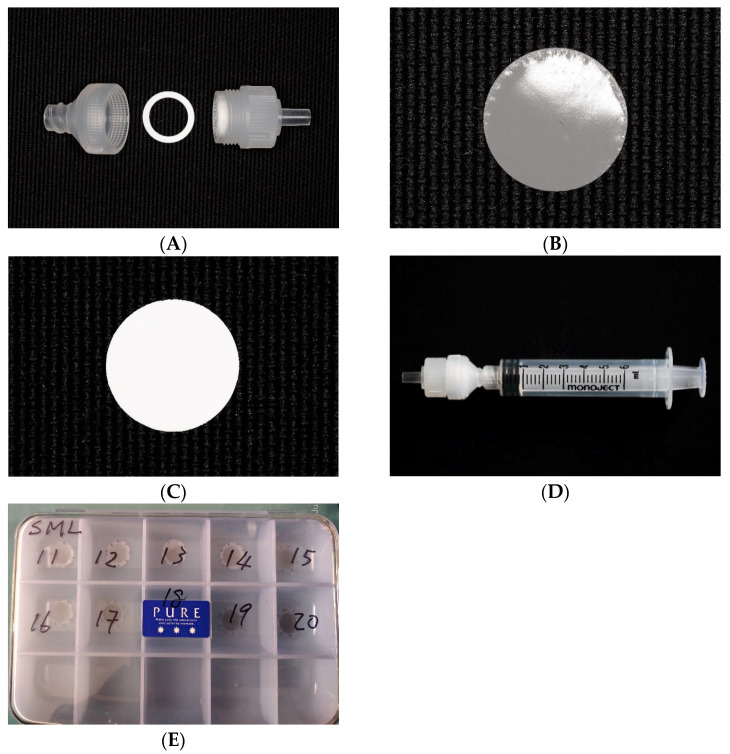
Filtration setup: Swinnex membrane holder, EMD Millipore Corporation (**A**), polycarbonate membrane, Isopore, Merck Millipore (**B**), nitrocellulose membrane, MF-Millipore, Merck Millipore (**C**), Swinnex assembled to a disposable 6 mL luer-lock syringe (**D**), sample collected and stored for SEM analysis (**E**).

Ten discs from each instrumentation group were assigned for particle size analysis under the SEM. The collected rinsing solution was subjected to membrane filtration to separate particles of interest from the solution. The membrane filtration setup consisted of a Swinnex membrane holder (Swinnex, EMD Millipore Corporation, Billerica, MA, USA) (Figure 6A) that housed two overlapping 13 mm membranes. A polycarbonate membrane with 0.4 μm pore size (Isopore, Merck Millipore Ltd., Tullagreen, Carrigtwohill, Ireland) (Figure 6B) was placed facing the recipient end of the holder. A second nitrocellulose membrane, with a pore size of 0.8 μm, was tucked underneath the polycarbonate membrane at the exit end (MF-Millipore, Merck Millipore Ltd., Tullagreen, Carrigtwohill, Ireland) (Figure 6C). The polycarbonate membrane provided a smooth surface that is ideal for SEM analysis and the nitrocellulose membrane served as a support to the polycarbonate membrane that not only prevented particles from clumping up at the pores but also aided in the overall sealing of the Swinnex holder. A pore size of 0.4 μm was chosen based on the smallest Ti particle size reported in biopsy studies of peri-implantitis subjects [33]. The collected rinsing solution was dispensed through the filtration membranes using a disposable 6 mL syringe that was connected to the Swinnex via a luer-lock mechanism (Figure 6D). One syringe was used per sample, and the filtering step was repeated until all the collected rinsing solution was filtered through. Upon completion of initial sample filtration, the empty Digitube was filled with 30 mL of MilliQ solution to re-suspend potential residual particles for the final filtration. The polycarbonate membrane was then retrieved and stored in an assortment box for SEM analysis (Figure 6E). 

#### 2.3.2. Scanning Electron Microscopy and Energy-Dispersive Spectroscopy

**Figure 7:**
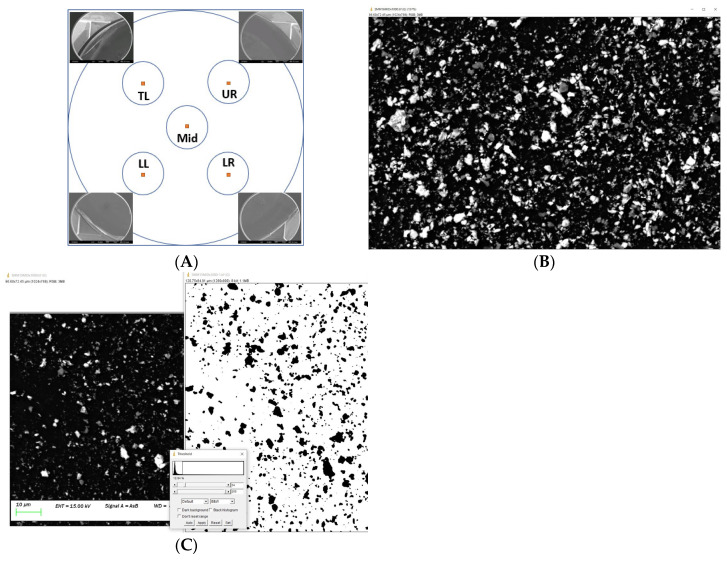
Area of interest for SEM analysis (**A**), data collection using ImageJ (**B**), and Ti particles represented in black over a white background (**C**).

The 13 mm polycarbonate membrane which contained particles of interest, was fixed on a stub with double-sided tape before being subjected to SEM analysis. Field emission SEM with energy-dispersive spectroscopy (Sigma VP FEG SEM, Zeiss, Oberkochen, Germany) was used to differentiate the observed particles and analyse their Feret’s diameter. There were five areas of interest on the polycarbonate membrane—top-left (TL), top-right (TR), middle (Mid), lower-left (LL), and lower-right (LR)—and this was standardised for data collection from each sample membrane. Four corners of the stub holder on the stage were used as the reference points for the areas of interest. Each area of interest was located 3 mm from each corner towards the centre of the membrane. The coordinates of the TL, TR, LL, and LR were used to calculate the 5th area of interest, Mid (Figure 7A). Five coordinates were then stored in the SEM viewing software for data collection at the same location on each sample membrane. The backscatter mode (Signal A (AsB)) was used to enhance the contrast of metal particles in the dark field. The brightness and contrast of the viewing software were set at 41% and 31%, respectively. An EDS analysis was carried out to identify particles that appeared differently in their contrast against the dark background. Two magnifications of micrographs, ×1000 and ×7500, were taken for particle size and particle number evaluation. 

#### 2.3.3. Data Collection and Interpretation

ImageJ software (ImageJ, version 1.53g, National Institutes of Health, Bethesda, MD, USA) was used to view and analyse the micrograph under ×1000 magnification. The 10 μm reference scale in the micrograph was used to set and calibrate the global scale in length (10.6 pixel/μm). Each area of interest consisted of a zone of 1280 × 900 pixels (120.75 × 84.91 μm) for particle analysis (Figure 7B). The image was then converted to 8-bit grayscale for threshold manipulation. Whilst the upper threshold level of the image was set at a maximum of 255, the lower threshold level was adjusted until all known non-Ti particles were eliminated by using images taken with the EDS as a reference. The Ti particles were presented in black over a white background (Figure 7C). Particle analysis was then carried out, both to count the number of particles and to measure the Feret maximum diameter of each particle, that is, the longest distance between any two points of a particle. Particles located on the edges were excluded in this analysis. 

### 2.4. Trace Element Analysis—Inductively Coupled Plasma Mass Spectrometry

Ten samples from each instrumentation procedure and control were used for ICP-MS Ti particle mass analysis. Samples collected in 50 mL Digitubes were evaporated to dry at 95 °C. The residues were then dissolved in 1 mL of certified hydrochloric acid (6N HCl) before diluting to 10 mL with MilliQ solution. The reconstituted sample was then diluted at a 1-to-10 ratio with 2% (*v*/*v*) nitric acid (HNO3) for analysis. A reference of internal standard was added for calibration to detect isotopes of Ti (^46^Ti, ^47^Ti, ^48^Ti, ^49^Ti, ^50^Ti). An Agilent 7900 quadrupole ICP-MS (Agilent Technologies, Inc., Santa Clara, CA, USA) was used to report the total Ti detected by mass (μg). Blank samples of each instrumentation group and control, that is, without contact with a Ti disc, were prepared and processed. All blank samples revealed a Ti mass below detection limits of 0.02 μg. The detected Ti mass from each group was subjected to statistical analysis.

### 2.5. Statistical Analysis

Statistical analysis was performed using the SPSS statistical software package (IBM SPSS statistics, version 27, Chicago, IL, USA). Descriptive statistics of Ti particle size, particle count, and Ti weight were presented in mean, ranges, and standard deviation (SD). The null hypothesis of data distributed normally was rejected by the Shapiro–Wilk test (*p* < 0.05); hence, data was logarithm-transformed to satisfy the assumption of normality before being analysed using a one-way analysis of variance (ANOVA) with pair-wise comparison. The significance level was set at *p* < 0.05 for all analyses. The principal investigator carried out data collection for SEM analysis of Ti particle size and particle counts. Repeated measurements on 20 micrographs were performed to assess intra-tester reliability with Cohen’s Kappa agreement. Scores of less than 0.4, 0.41–0.60, 0.61–0.80, and above 0.81 indicate poor, moderate, good, and almost perfect agreement, respectively [34].

## 3. Results

### 3.1. Scanning Electron Microscopy and Energy-Dispersive Spectroscopy

#### 3.1.1. Ti Disc and Instruments

**Figure 8:**
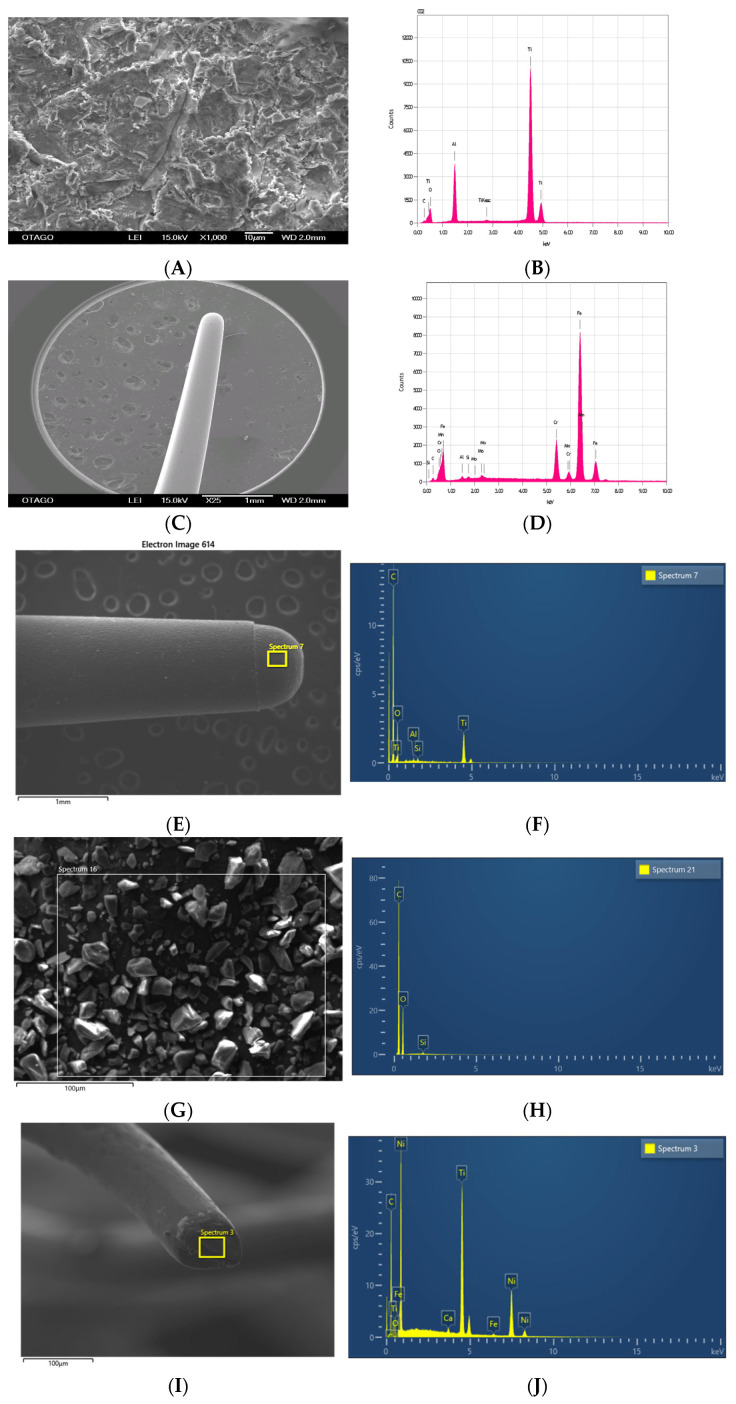
Chemical composition of Ti disc and instruments under ×1000 magnification: moderately roughened Ti disc (**A**,**B**), ultrasonic metal tip (**C**,**D**), ultrasonic PEEK tip (**E**,**F**), erythritol powder (**G**,**H**), and Ti brush (**I**,**J**).

SEM and EDS analysis were conducted on pristine moderately roughened Ti discs, ultrasonics tips, erythritol powder, and Ti brushes prior to sample collection. Under ×100 magnification, the aluminium-oxide-blasted Ti surface showed random irregularities with various degrees of ridges and valleys. EDS analysis revealed surface elements consisting of mainly Ti, aluminium (Al), and oxygen (O) (Figure 8A,B). Both metal and PEEK tips appeared smooth on the surface under magnification. Metal elements such as iron (Fe), chromium (Cr), manganese (Mn), molybdenum (Mo), and Al were found to be the predominant composition of the metal tip, whereas carbon (C), O, and Ti dominated the PEEK high-plastic tip (Figure 8C–F). The composition of erythritol powder was predominately composed of C, O, and silicon. In contrast to the product description, the largest particle observed under the SEM was significantly larger than was advised by the manufacturer (i.e., 38 μm and 14 μm) (Figure 8G,H). Elements such as Ti, nickel (Ni), Fe, C, and O were identified on the bristle of the NiTi-based Ti brush that was 0.1 mm in diameter (Figure 8I,J). 

#### 3.1.2. Particle Identification—Energy-Dispersive Spectroscopy

##### Metal Tip Ultrasonic Scaling 

**Figure 9:**
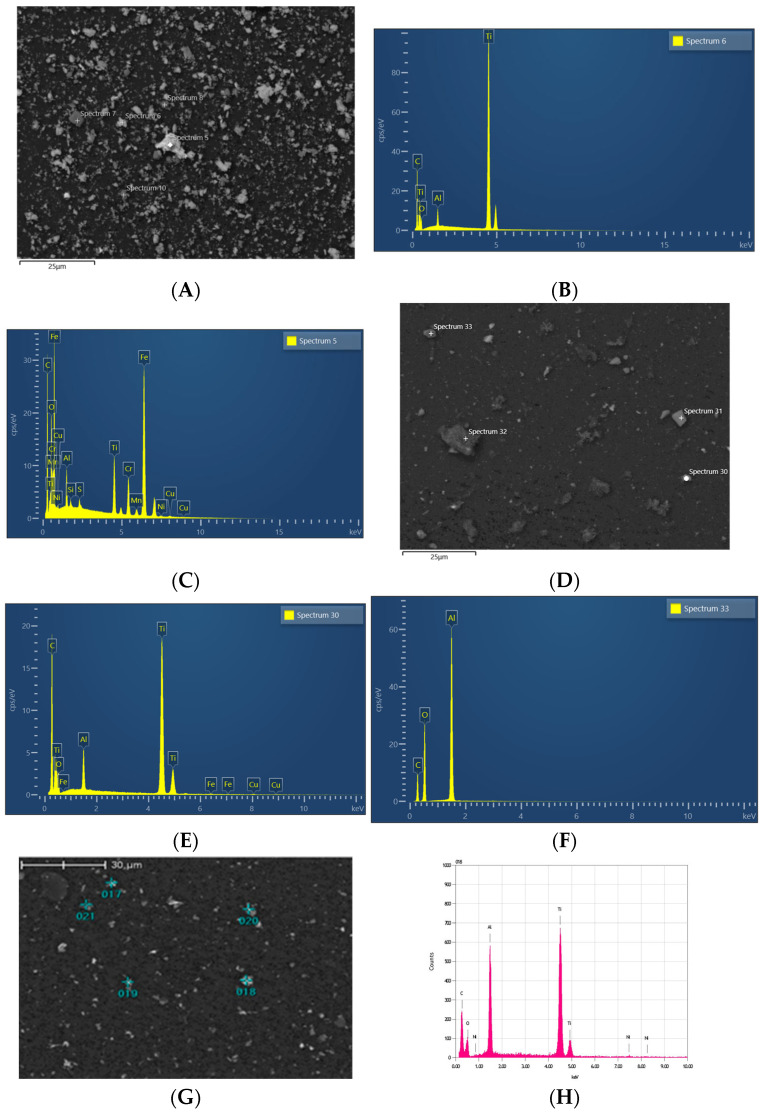
Particles identified from mechanical decontamination procedures: metal tip ultrasonic scaling under ×1000 magnification (**A**), spectrum 6, Ti (**B**), spectrum 5, Fe, Cr (**C**); PEEK tip ultrasonic scaling under ×1000 magnification (**D**), spectrum 30, Ti (**E**), spectrum 33, Al, C (**F**); Ti Brush under ×1000 magnification (**G**), spectrum 17, Al, Ti (**H**).

Under SEM observation in backscatter mode, metal particles with irregular shapes and of various sizes were observed in both metal ultrasonic scaling groups (Figure 9A). These metal particles appeared as different levels of contrast, representing different component elements, against the black background. The EDS analysis revealed the particles to be Fe, Ti, and Al (Figure 9B,C). 

##### PEEK Tip Ultrasonic Scaling

The particles identified in the two ultrasonic scaling PEEK groups were Ti, Al, C, and Si (Figure 9D–F). Of the elements present, Ti stood out as having the most contrast. Residues of the PEEK tip could be observed under SEM observation as islands of collected submicron particles, which EDS identified as C, O, Ti, and Si. Due to the presence of organic elements, the Ti within the particle islands appeared to be a lower contrast (darker) than the larger isolated Ti particles identified. 

##### Ti Brush

The particles observed in the Ti brush group closely resembled the ultrasonic scaling metal group but were lesser in quantity (Figure 9G,H). Traces of either Al or Ni could be detected on the Ti particles identified, suggesting that the Ti particles originated from the treated Ti disc or from the NiTi bristle of the Ti brush, respectively.

##### Erythritol Air-Polishing

The polycarbonate membranes from the air-polishing group retained particles of interest as well as retaining a thick layer of erythritol powder residue. Because it was impossible to isolate the Ti particles from the powder residues, SEM analysis was conducted on the remnants retained on the polycarbonate membrane after removing the mass of powder residues. Ti particles were occasionally identified in the areas of interest, surrounded by aggregations of powder material that consisted predominately of Si and organic elements such as C and O.

##### Control

In the control group, very few metal particles were found on the polycarbonate membranes, and these were Ti. 

#### 3.1.3. Particle Analysis

Under a magnification of 1000, non-Ti particles such as Al were eliminated from the area of interest by adjustment of a lower threshold level in the ImageJ software. (ImageJ, version 1.53 g, National Institutes of Health, USA). The exclusion of Fe was not possible because of its similarity in level of contrast to Ti. Furthermore, islands of particles containing Ti that were observed in the ultrasonic scaling PEEK group could not be included because of the low level of contrast under a magnification of 1000. 

##### Size of Released Ti Particles

**Figure 10:**
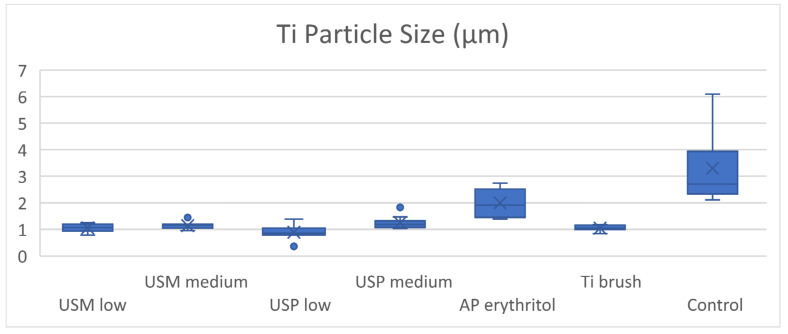
Boxplot of the mean Ti particle size (μm).

The mean diameter of the metal particles collected from the metal tip ultrasonic scaling groups at low and medium power settings were 1.06 ± 0.16 μm and 1.15 ± 0.14 μm, respectively. In contrast, the PEEK ultrasonic scaling groups produced particles of 0.89 ± 0.27 μm and 1.25 ± 0.24 μm in size at low and medium power settings, respectively. No significant difference was found in the mean diameter of particles generated by any of the ultrasonic scaling groups, except for ultrasonic scaling using the PEEK tip, where a significantly smaller particle diameter was found at the low power setting compared to the medium power setting. Furthermore, the Ti brush generated particles with a mean diameter of 1.05 ± 0.27 μm, which was not significantly different from all of the ultrasonic scaling groups. Upon removing the mass of powder residues, the remaining Ti particles found on the polycarbonate membrane were significantly larger than other test groups at 2.00 ± 0.54 μm. Particles dislodged from the surface of the Ti disc by air–water spray in the active control group were significantly larger than all the test groups at 3.30 ± 1.28 μm (Table 1, Figure 10).

##### Quantity of Released Ti Particles—Particle Count

**Figure 11:**
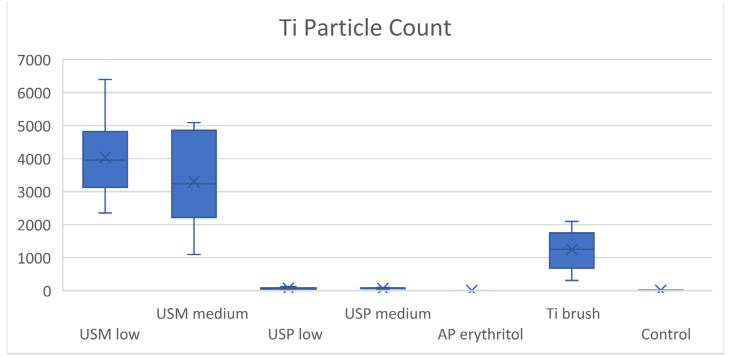
Boxplot of mean Ti particle count.

The metal-tip ultrasonic group produced a significantly higher number of particles than other types of instrumentation. At low and medium power settings, 4043.40 ± 1196.96 and 3306.40 ± 1196.96 particles were generated in 20 s, respectively. The Ti brush group produced 1246 ± 584.04 particles, and this was significantly more than the ultrasonic scaling with the PEEK tip at both low (76.60 ± 28.54) and medium power (67.80 ± 23.20), as well as air-polishing (7.50 ± 1.43) and the control group (13.50 ± 7.89). The Ti particle count in the air-polishing group was the lowest compared to all test groups and the control group. No significant difference in particle count was found between the same ultrasonic scaling groups of different power settings (Table 2, Figure 11). 

### 3.2. Trace Element Analysis—Inductively Coupled Plasma Mass Spectrometry

The quantity of Ti in the rinsing solution was also analysed by ICP-MS, which provided the total mass of Ti as the quantitative measure. In comparison to the particle count for Ti, descriptive statistics and the boxplots demonstrated similarity between the two outcome measurements, except for the air-polishing and control groups where the air-polishing group generated significantly more Ti in the rinsing solution (0.67 ± 0.12 μg) compared to the control group (0.19 ± 0.14 μg). 

**Figure 12:**
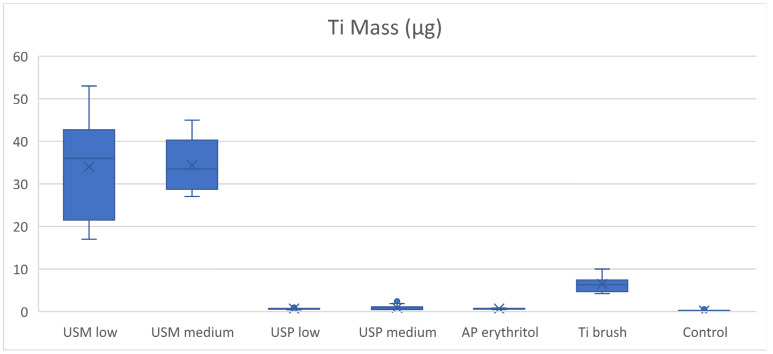
Boxplot of Ti mass (μg).

Twenty seconds of ultrasonic scaling with the metal tip was capable of generating 34.00 ± 12.54 μg and 34.44 ± 12.54 μg of Ti at low and medium power settings, respectively. Significantly less Ti (0.66 ± 0.14 and 0.89 ± 0.14 μg at low and medium power settings, respectively) was produced during ultrasonic scaling using the PEEK tip. No significant difference in Ti weight was found between the same ultrasonic scaling groups of different power settings. The Ti brush produced 6.45 ± 2.03 μg of Ti, which was significantly less than the metal-tip ultrasonic scaling groups but significantly higher than all other instrumentation methods (Table 3, Figure 12) 

## 4. Discussion

### 4.1. Size of Ti Particles

The in vitro particle analysis undertaken for this research project demonstrated that commonly applied mechanical decontamination procedures can generate different amounts of Ti particles of various sizes from moderately roughened Ti discs. Overall, the mean size of Ti particles did not vary significantly among the test groups, except for the erythritol air-polishing, where the particles were much larger. Variables such as whether the ultrasonic scaler was metal or PEEK and whether the power setting was low or medium did not result in a significant difference in the size of the generated Ti particles.

Ultrasonic scaling using the PEEK tip can generate islands of various sizes that contain elements of C, O, Ti, and Si. The elemental composition is identical to the elemental profile identified on the PEEK tip; therefore, it is reasonable to suggest that deterioration of the PEEK tip can release an additional amount of Ti to the environment. However, size analysis of the particles within the island was not possible due to the co-existence of organic elements that resulted in a low contrast in appearance. 

The EDS detected a trace of either Al or Ni on the Ti particles released during rotary Ti brush instrumentation. The presence of Al strongly suggests the Ti particles to be derived from the aluminium-oxide-blasted moderately roughened Ti surface, and the presence of Ni suggests that the observed Ti particles originated from the NiTi bristle of the Ti brush. The flattened bristle tip observed under SEM indeed confirmed that the deterioration of a NiTi brush can provide an additional route of Ti release during mechanical instrumentation. 

There is a lack of studies that evaluate the characteristics of Ti particulates released during mechanical decontamination procedures in the current literature. To date, almost all of the research available in this area is limited to ultrasonic scaling. The current study is one of the first Ti particulate analyses that included other decontamination procedures, such as Ti brushing or air-polishing. In an in vitro study, Eger and colleagues analysed the size of metal particles generated during ultrasonic scaling with a metal tip on two implant surfaces [19]. They reported mean particle sizes of 7.57 ± 2.75 μm and 8.37 ± 2.94 μm on SLA and SB Ti surfaces, respectively, which were larger compared to the findings of the current study. The variation in the reported particle size could be caused by differences in the methodology of the two studies. Although both Eger’s and the current study used moderately roughened Ti surfaces, the Eger study used Ti discs made of a Ti6Al4V Ti alloy, whereas the current study used commercially pure Ti. The hardness of the Ti could have potentially not only affected the characteristics of the particle shedding from the treated surface, but also caused a higher rate of deterioration of the instrument. Apart from the difference in the type of Ti that Eger and colleagues [19] used in 2017, the scaling protocol they used was 60 s of ultrasonic scaling. This resulted in an additional 40 s of ultrasonic scaling compared to the current study, with a progressively deteriorated metal instrument against the progressively smoother Ti surface. Similarly, ultrasonic scaling, which was conducted on a custom-made platform in the current study, resulted in repetition of the instrumentation in one single direction. A worn instrument activated against a smoother surface could have potentially resulted in generating Ti particles that were less representative of a clinical scenario.

The mean particle sizes reported by the current study fall within the threshold size of 10 μm, below which the particles can be engulfed by macrophages, neutrophils, fibroblasts, and osteoblasts [20,35,36,37]. This suggests that there is an untapped a potential route of promoting inflammation and the upregulation of MMP production in the peri-implant niches [22,38]. In addition, this finding is also in line with the size of Ti particles (1 to 3 μm) found within macrophages that were isolated from retrieved failed implants [33]. Human biopsy studies of soft tissue with peri-implantitis reported Ti particles found between 2 and 54 μm [39,40]. In this stage of disease, the deposition of Ti particles into the peri-implant soft tissue can be the result of several mechanisms, including osteotomy and implant surgery during the placement, stage-two surgery or restorative procedures, functional loading, bio-tribocorrosion, and mechanical debridement during treatment or early maintenance visits. The reported wide range of particle sizes thus represents the history of the implant throughout its life span. 

Biopsy and cytology smear studies provide cross-sectional findings that describe the characteristics of Ti particles at the time tissue samples were collected. The early presence of Ti particles was identified from tissue samples taken during implant placement and stage-two surgery in both human and animal studies, representing the superficial Ti particles that were dislodged as the result of frictional and torsional force created on the implant surface during implant insertion. Flatebo and colleagues [41] reported Ti particle sizes ranging from 0.14 to 2.3 μm, and Tanaka and colleagues [42] identified particle sizes of 1.8 to 3.2 μm in biopsies taken from beagle dogs. These reported ranges of particle sizes were similar to the significantly large particles of 3.30 ± 1.28 μm observed in the active control group of the current study. It can reasonably be assumed that air–water spray at 350 kPa dislodged the loosely attached Ti particles from the pristine, moderately roughened Ti surface. 

### 4.2. Quantity of Ti Particles

Significantly different amounts of Ti particles were observed across different forms of mechanical instruments. Generally, the metal form of instruments generated a higher amount of Ti than the non-metal forms. Metal-tip ultrasonic scaling released significantly higher amounts of Ti from the Ti discs in comparison to their PEEK counterparts. Within the same forms of ultrasonic scaling, no significant difference was found between the two different power settings. The Ti brush generated a significantly lower level of Ti when compared to the metal ultrasonic group; however, it was also significantly higher than both ultrasonic scaling PEEK groups, as well as the erythritol air-polishing group. Significantly less in number but larger in size Ti particles were observed in the control group where the Ti discs were treated by air–water spray.

Tawse-Smith and colleagues [18] conducted an in vitro analysis on Ti surfaces that were altered by ultrasonic scaling with metal and PEEK tips. Lower mean Ti masses of 11.13 ± 7.60 μg and 0.18 ± 0.13 μg were reported in the adjunctive ICP-MS analysis of the rinsing solution collected from the metal and PEEK tip groups, respectively. The same ultrasonic device and similar instrumentation were deployed in this study as Tawse-Smith and colleagues used in their 2016 research [18]. The angulation and the force applied during instrumentation were standardised by using a custom-made platform. Furthermore, the rinsing solution was collected within an enclosed container, followed by standardised 10 s (approximately 15 mL) post-instrumentation rinsing to retrieve particles from the platform.

In an animal study, Wang and colleagues [26] reported a significantly high level of peri-implant macrophage infiltration, accompanied by a significantly increased peri-implant bone loss, in the presence of 20 μg Ti particles with a mean size of 3.32 ± 2.39 μm. Metal-tip ultrasonic scaling, regardless of power setting, was capable of generating higher amounts of smaller Ti particles that were more reactive to living cells [20,22,24,43] than the Ti particles used in Wang et al.’s animal study. A linear inverse relationship between the Ti concentration and the viability of rat osteoblasts was observed by Pioletti et al. (1999) when the Ti concentration was higher than 1.5 mg/mL (3.1 ± 3.6 μm) [25]. The suggested Ti concentration threshold is significantly higher than the amount that can be generated by a single session of 20 s of mechanical debridement. It is therefore reasonable to interpret that the cytotoxic effect of Ti particles on an osteoblast can only take place when the peri-implant Ti particles accumulate without disturbance over time.

Quantification of Ti released during decontamination procedures has also been evaluated by direct Ti particle count. Boxplots of the results show a similarity in the distribution of the quantity of Ti among the groups when compared to the Ti mass analysed by ICP-MS. Although this outcome measurement allows for inter-group comparison within a study, it is difficult to compare the results between different studies if the total Ti particle count and instrumentation time were not reported. In the current study, the sum of Ti particles counted from the five fields of interest (120.75 × 84.91 μm) per sample was reported for inter-group comparison. The total number of Ti particles per sample can only be estimated from the total surface area of the 133 mm2 membrane; hence, the mean Ti particle counts of 4043 ± 1197 and 3306 ± 1352 reported for metal-tip ultrasonic scaling at the low and medium power settings can be estimated at a total of 10 and 8.8 million Ti particles generated in 20 s of instrumentation, respectively. Despite the effort made to exclude non-Ti metal particles, this estimation of the total Ti particle count is higher than the 3.6 million metal particles reported by Eger et al. [19], who observed 60 s of metal-tip ultrasonic scaling, as well as the 3889 mean total metal particles reported by Harrel and colleagues (2019) [44]. The major limitation of direct particle count in the quantification of Ti released during surface decontamination procedures is the inability to isolate Ti from the other metal particles. On this basis, ICP-MS analysis of the overall Ti mass appears to be the superior method over a direct Ti particle count.

To date, available evidence that reports the physicochemical properties of Ti released during mechanical decontamination methods is limited solely to ultrasonic scaling. The current study demonstrated that a Ti brush can generate 6.45 ± 2.03 μg of Ti (1246 ± 584 particle count) during 20 s of instrumentation, which is significantly less than the ultrasonic scaling metal-tip groups. SEM and EDS observation also suggested that the origin of the released Ti is not limited to the instrumented moderately roughened Ti surface, but also comes from the Ti brush that shows deterioration at the tip of NiTi-containing bristles after instrumentation. A similar observation was made on the PEEK tip. Ti was identified in the elemental composition of the instrument, and this is in line with the finding reported by Tawse-Smith and colleagues [18], providing an additional source of Ti release. A significantly lower amount of Ti was generated by the PEEK tip (0.66 ± 0.14 μg and 0.89 ± 0.68 μg; 75 ± 29 and 68 ± 23 particles counted at the low and medium power settings, respectively) and the erythritol air-polishing groups (0.67 ± 0.12 μg; 8 ± 1 particle count) when compared to all powered metal instruments. No significant difference was found in the reported mass of Ti between these non-metal instrumentation groups; however, a significantly lower Ti particle count was found in the air-polishing group in comparison to the PEEK groups. This can be explained by the significantly larger Ti particles observed in the air-polishing group and the presence of powder residues that prevented the disclosure of Ti particles under SEM observation.

The current in vitro study demonstrated that air-polishing with erythritol powder could result in the collection of power residues in the instrumented environment. However, the minimum residual particles observed on the treated Ti surface under SEM also suggest that additional rinsing is adequate to remove the particle deposition. Because of this, the effect of powder residues on the biocompatibility of Ti surfaces could be minimal and comparable to the pristine status as suggested by the systematic review conducted by Louropoulou and colleagues [5]. Biocompatible, non-cariogenic, and non-toxic natural sugar alcohol erythritol in combination with chlorhexidine has been introduced to the market in the recent years. Higher post-treatment biofilm re-growth inhibition was observed, in comparison to air-polishing using sodium bicarbonate powder, because of its ability to alter the microstructure and metabolism of the P. gingivalis-dominated biofilm, which had the addition of chlorhexidine that provides an additional antibiofilm benefit [45].

### 4.3. Limitations of the Current Study

Post-instrumentation SEM observation revealed flattening of the instrumented moderately roughened Ti surface as well as deterioration of the metal tip. The final few repeated strokes of ultrasonic scaling might have represented the effect of instrumentation on the smooth Ti surface. Whether a multi-directional instrumentation setup can better represent a clinical situation and reflect potential effects of different ultrasonic power settings is an interesting question and one that requires further investigation.

A membrane with a 0.4 μm pore size was selected for the current study, and this was based on the smallest particle size of Ti reported by biopsy studies [29]. Even so, Ti particles that are smaller than 0.4 μm can still be observed under ×7500 magnification. Particle size analysis under ×1000 magnification allows for a general overview of the size distribution, but the smallest particle that could be measured under this field of view was 0.364 μm. A limitation in this research, therefore, lies in detecting particles that are smaller than 0.4 μm.

Furthermore, membrane filtration of the powder containing rinsing solution collected from the erythritol air-polishing group resulted in an accumulation of powder residues, and isolation of the Ti particles from the powder residues was not possible. Because of this, the reported mean particle size of Ti and the particle count by the SEM analysis could only represent the Ti particles that remained on the polycarbonate membrane after the removal of most powder deposits. The ICP-MS Ti mass analysis of the air-polishing samples shared a similar shortcoming.

In addition, the findings of this in vitro study could serve as a foundation for future research.

1. The reported size and quantity of Ti from the current study can be applied in future in vitro and in vivo analyses to identify the potential role of Ti released during mechanical decontamination procedures.
2. It has been reported in the literature and by the current study that metal instruments can also generate other non-Ti metal particles. The quantity, as well as the potential pro-inflammatory and cellular response, of non-Ti metal particles should be evaluated to aid in justifying the instrument of choice in the clinical setting.

## 5. Conclusions

Within the limitations of the current study, it can be concluded that Ti surface mechanical decontamination procedures can produce Ti particulates and potentially modify surfaces to varying degrees. Ultrasonic scaling using a metal tip generates the highest amount of Ti with small-sized Ti particles when compared to all other commonly used mechanical surface instrumentations. The significantly large-sized and low levels of Ti particles reported in air-polishing with erythritol powder should be interpreted with caution due to the limitation of potential contamination of the samples from the powder residues. The EDS analysis confirmed the presence of Ti in the PEEK tip samples. And on this basis, it can be suggested that the deterioration of the PEEK tip and Ti brush, as observed under SEM, is an additional source of Ti release during mechanical debridement.

Currently, matured biofilm is an aetiological factor of peri-implant diseases; therefore, cause-related therapy, involving decontamination procedures to remove the bacterial biofilm and restore homeostasis, is the mainstream treatment. Powered metal instruments, such as ultrasonic scaling, are the most effective and efficient ways to achieve the above desired outcome. However, Ti surface alteration and Ti particulate release are inevitable and should be expected during the mechanical decontamination of infected implant surfaces. Furthermore, the size of Ti particles and the amount of Ti particulates generated during commonly applied mechanical decontamination methods fall within the pro-inflammatory threshold suggested by the current literature. Air-polishing with erythritol powder formula is also capable of generating Ti particles of a bioreactive size. However, the quantity of Ti generated is significantly less than other tested metal instruments. This finding, in addition to the superior post-instrumentation surface biocompatibility of erythritol powder air-polishing reported in the literature, may provide additional benefits in surface decontamination prior to defect reconstruction in managing peri-implantitis.

## Figures and Tables

**Table 1:** Comparison of mean diameter (μm) of the Ti particles released from Ti discs during different mechanical decontamination methods. * Significant difference between the two compared groups (*p* value < 0.05) by one-way ANOVA pair-wise comparison. Note: AP: air-polishing; SD: standard deviation; Ti: titanium; USM: ultrasonic scaling metal tip; USP: ultrasonic scaling PEEK tip.

Instrumentation	Sample Size	Mean Diameter (SD) (μm)	Comparison against	Mean Difference (μm)	SE	Sig. *
USM Low	10	1.06 (0.16)	USM Medium	0.09	0.106	*p* = 0.98
			USP Low	0.17	0.106	*p* = 0.46
			USP Medium	−0.19	0.016	*p* = 0.71
			AP Erythritol	−0.94	0.106	*p* < 0.05 *
			Ti Brush	0.01	0.106	*p* = 1.00
			Control	−2.24	0.109	*p* < 0.05 *
USM Medium	10	1.15 (0.14)	USP Low	0.26	0.106	*p* = 0.11
			USP Medium	−0.10	0.106	*p* = 0.99
			AP Erythritol	−0.85	0.106	*p* < 0.05 *
			Ti Brush	0.10	0.106	*p* = 0.98
			Control	−2.15	0.109	*p* < 0.05 *
USP Low	10	0.89 (0.27)	USP Medium	−0.36	0.106	*p* < 0.05 *
			AP Erythritol	−1.11	0.106	*p* < 0.05 *
			Ti Brush	−0.16	0.106	*p* = 0.50
			Control	−2.41	0.109	*p* < 0.05 *
USP Medium	10	1.25 (0.24)	AP Erythritol	−0.75	0.106	*p* < 0.05 *
			Ti Brush	0.20	0.106	*p* = 0.68
			Control	−2.05	0.109	*p* < 0.05 *
AP Erythritol	10	2.00 (0.54)	Ti Brush	−0.75	0.106	*p* < 0.05 *
			Control	−2.05	0.109	*p* < 0.05 *
Ti Brush	10	1.05 (0.11)	Control	−2.25	0.109	*p* < 0.05 *
Control	10	3.30 (1.28)	-	-	-	-

**Table 2:** Comparison of mean number of Ti particles released from Ti discs during different mechanical decontamination methods. * Significant difference between the two compared groups (*p* value < 0.05) by one-way ANOVA pair-wise comparison. Note: AP: air-polishing; SD: standard deviation; Ti: titanium; USM: ultrasonic scaling metal tip; USP: ultrasonic scaling PEEK tip.

Instrumentation	Sample Size	Mean Particle Count (SD)	Comparison against	Mean Difference	SE	Sig. *
USM Low	10	4043 (1197)	USM Medium	737	0.197	*p* = 0.86
			USP Low	3968	0.197	*p* < 0.05 *
			USP Medium	3975	0.197	*p* < 0.05 *
			AP Erythritol	4035	0.197	*p* < 0.05 *
			Ti Brush	2797	0.197	*p* < 0.05 *
			Control	4030	0.202	*p* < 0.05 *
USM Medium	10	3306 (1352)	USP Low	3231	0.197	*p* < 0.05 *
			USP Medium	3238	0.197	*p* < 0.05 *
			AP Erythritol	3298	0.197	*p* < 0.05 *
			Ti Brush	2060	0.197	*p* < 0.05 *
			Control	3293	0.197	*p* < 0.05 *
USP Low	10	75 (29)	USP Medium	7	0.197	*p* = 1.00
			AP Erythritol	67	0.197	*p* < 0.05 *
			Ti Brush	−1171	0.197	*p* < 0.05 *
			Control	62	0.202	*p* < 0.05 *
USP Medium	10	68 (23)	AP Erythritol	60	0.197	*p* < 0.05 *
			Ti Brush	−1178	0.197	*p* < 0.05 *
			Control	55	0.202	*p* < 0.05 *
AP Erythritol	10	8 (1)	Ti Brush	−1238	0.197	*p* < 0.05 *
			Control	−5	0.202	*p* = 0.06
Ti Brush	10	1246 (584)	Control	1233	0.202	*p* < 0.05 *
Control	10	13 (8)	-	-	-	-

**Table 3:** Comparison of the mean Ti mass released from Ti discs during different mechanical decontamination methods. * Significant difference between the two compared groups (p value < 0.05) by one-way ANOVA pair-wise comparison. Note: AP: air-polishing; SD: standard deviation; Ti: titanium; USM: ultrasonic scaling metal tip; USP: ultrasonic scaling PEEK tip.

Instrumentation	Sample Size	Mean Ti Mass (SD) (μg)	Comparison against	Mean Difference (μg)	SE	Sig. *
USM Low	10	34.00 (12.54)	USM Medium	−0.40	0.182	*p* = 1.00
			USP Low	33.34	0.182	*p* < 0.05 *
			USP Medium	33.11	0.182	*p* < 0.05 *
			AP Erythritol	33.33	0.182	*p* < 0.05 *
			Ti Brush	27.55	0.182	*p* < 0.05 *
			Control	33.81	0.182	*p* < 0.05 *
USM Medium	10	34.40 (6.08)	USP Low	33.74	0.182	*p* < 0.05 *
			USP Medium	33.51	0.182	*p* < 0.05 *
			AP Erythritol	33.73	0.182	*p* < 0.05 *
			Ti Brush	27.95	0.182	*p* < 0.05 *
			Control	34.21	0.182	*p* < 0.05 *
USP Low	10	0.66 (0.14)	USP Medium	−0.23	0.182	*p* = 0.99
			AP Erythritol	−0.01	0.182	*p* = 1.00
			Ti Brush	−5.79	0.182	*p* < 0.05 *
			Control	0.47	0.182	*p* < 0.05 *
USP Medium	10	0.89 (0.68)	AP Erythritol	0.22	0.182	*p* = 1.00
			Ti Brush	−5.56	0.182	*p* < 0.05 *
			Control	0.70	0.182	*p* < 0.05 *
AP Erythritol	10	0.67 (0.12)	Ti Brush	−5.78	0.182	*p* < 0.05 *
			Control	0.48	0.182	*p* < 0.05 *
Ti Brush	10	6.45 (2.03)	Control	6.26	0.182	*p* < 0.05 *
Control	10	0.19 (0.14)	-	-	-	-

## Data Availability

The data presented in this study are available on request from the corresponding author.
